# On-Resin Acetamidomethyl (Acm) Removal and Disulfide Formation in Cysteinyl Peptides Using *N*-Chlorosuccinimide (NCS) in the Presence of Other Cys-Protecting Groups

**DOI:** 10.3390/ijms26062523

**Published:** 2025-03-11

**Authors:** Amit Chakraborty, Fernando Albericio, Beatriz G. de la Torre

**Affiliations:** 1 Peptide Science Laboratory, School of Chemistry and Physics, University of KwaZulu-Natal, Westville, Durban 4000, South Africa; amit24816@gmail.com; 2 Department of Organic Chemistry, University of Barcelona, 08028 Barcelona, Spain; 3 School of Laboratory Medicine and Medical Sciences, College of Health Sciences, University of KwaZulu-Natal, Durban 4041, South Africa

**Keywords:** Cys, disulfide bridge, protecting groups, SIT, solid-phase peptide synthesis

## Abstract

Acetamidomethyl (Acm)-protected cysteine derivatives are essential components of multi-disulfide synthesis, particularly due to the availability of multimodal removal conditions for Acm protection. Most of these removal conditions are harsh and are commonly used to remove Acm protection at the last step of regioselective synthesis of a multi-disulfide, implying that the removal of Acm is performed in the absence of other Cys thiol protections. In this context, *N*-chlorosuccinimide (NCS)-mediated removal of Acm and concomitant disulfide bridge formation provides a fast and reliable way to synthesize multi-disulfides. In the present study, we demonstrate that NCS-mediated Acm removal and disulfide bond formation can be performed in the presence of other commonly used Cys thiol protections. Interestingly, Acm can be removed with NCS without affecting the Trt group, which is also removed with I_2_. Later, we successfully employ the NCS-based Acm removal method in the synthesis of multi-disulfide peptides like α-conotoxin SI.

## 1. Introduction

Peptide drug development has seen phenomenal progress in the last couple of decades [1,2,3]. Generally, peptides have a molecular weight of ≤5000 and offer advantages such as specificity, low toxicity, and predictable metabolism, among others [4,5]. The increasing influence of peptide therapeutics is evident from the FDA (U.S. Food and Drug Administration) approval of >100 peptide drugs [6,7]. A significant portion of these peptide drugs contains either cysteine (Cys) or disulfide bonds, which are formed by bridging two Cys side-chain thiols [8,9]. For example, Linaclotide, a 14-mer peptide with three disulfide bridges, is used for the treatment of irritable bowel syndrome [10,11]. It is worth mentioning that the disulfide bond in peptides enhances pharmacological properties such as prolonged stability and increased activity in a broad range of physiological conditions [12,13]. Additionally, the unique 3D conformation observed in disulfide-bridged peptide drugs improves membrane permeability and absorption of the drug [14,15]. Chemical synthesis of these disulfide-bridged peptides is always challenging. The synthesis of disulfide-rich peptides with desired disulfide connections is achieved using solid-phase peptide synthesis (SPPS) protocols in combination with solution-phase techniques [16,17]. 

Regioselective/stepwise synthesis of multi-disulfide peptides employing the SPPS technique requires the use of a combination of different Cys residues. Side-chain thiols in these Cys residues are protected with different protecting groups (PGs) [18,19]. Most PGs, like trityl (Trt), 4-methoxytrityl (Mmt), diphenylmethyl (Dpm), etc., are labile to acidic condition, a prerequisite to fit with SPPS protocols—to be precise, with Fmoc/*t*Bu-based SPPS protocols. There are other PGs, such as *tert-*butyl mercaptan (S*t*Bu), *sec*-isoamyl mercaptan (SIT), acetamidomethyl (Acm), phenylacetamidomethyl (Phacm), etc., that require removal conditions using reducing agents, base heavy metals like palladium, and even enzymes. Among this vast pool of Cys thiol PGs, Acm is quite unique with its multimodal removal conditions [20,21]. Acm is stable to Fmoc-SPPS protocols, is compatible with almost all other Cys thiol PGs, and can be removed with several conditions, including iodine [22,23], Pd(II) [24], DTNP [2,2′-dithiobis (5-nitropyridine)] in TFA/thioanisole [25], and *N*-halosuccinimide [26,27]. Surprisingly, Acm can also be removed with prolonged treatment of TIS/TFA (2/98) [28,29]. Most of these removal conditions facilitate Acm group removal and concomitant disulfide bond formation between two Acm-protected Cys thiols. During the stepwise synthesis of multi-disulfide peptides, it is customary to remove Acm protection at the last step to form the final disulfide bond. In this context, the use of *N*-halosuccinimide affords removal of Acm with fast reaction kinetics. The use of *N*-iodosuccinimide is reported to afford one-pot on-resin removal of Acm and disulfide bond formation in Cys(Acm) peptides [26,30]. Recently, *N*-chlorosuccinimide (NCS) and *N*-bromosuccinimide (NBS) were employed for one-pot regioselective synthesis of disulfide bonds in Cys(Acm) peptides under mild aqueous conditions [27]. In the present study, we demonstrate one-pot on-resin removal of Acm and disulfide bond formation in Cys(Acm) peptides using NCS. Furthermore, this transformation (Acm removal and disulfide bond formation from two Acm) can be carried out in the presence of other residues of Cys protected with different protecting groups such as Trt and SIT, which facilitates the regioselective formation of disulfide bridges.

## 2. Results and Discussion

**Figure 1:**
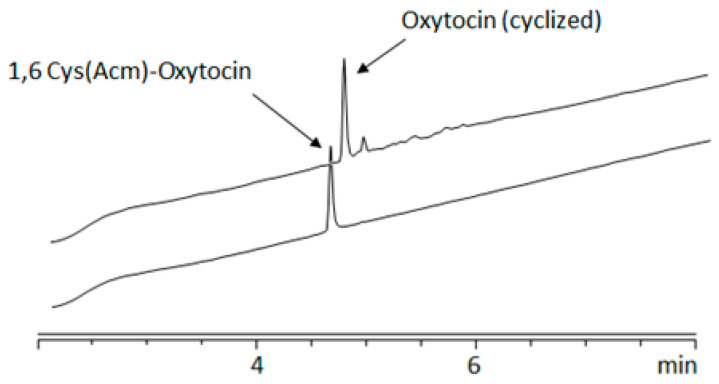
HPLC chromatograms of the NCS-mediated cyclization of Acm-protected oxytocin.

On-resin oxidation of bis-cysteinyl peptide using NCS is rapid and finishes very quickly. In our previous studies, NCS-mediated oxidation of free thiols to form a disulfide bond was achieved in just 15 min using 2 equiv. of NCS [31,32]. Given this, we hypothesized that the NCS-mediated removal of Acm from Cys thiol and the simultaneous formation of a disulfide bond could also be achieved quickly, using a higher equivalence of NCS. To test this, we attempted the on-resin cyclization of protected linear oxytocin resin [H-Cys(Acm)-Tyr(*t*Bu)-Ile-Gln(Trt)-Asn(Trt)-Cys(Acm)-Pro-Leu-Gly-NH-Rink amide AM resin], a well-known model therapeutic nonapeptide used to validate disulfide formation strategies. Linear oxytocin has two Cys residues at positions 1 and 6, which are connected by a disulfide bridge when cyclized. Initially, the linear nonapeptide [1,6-Cys(Acm)] was synthesized on Fmoc-Rink amide AM resin following a standard Fmoc/*t*Bu-based SPPS protocol. The quality of the synthesis was confirmed using the crude obtained after drying and cleaving a small amount of the nonapeptide resin. HPLC and LCMS analysis indicated the formation of an Acm-protected linear peptide with >95% purity (Figure 1 and Figure S2 in Supplementary Materials). For on-resin cyclization, the peptidyl resin (0.05 mmol) was treated with 3 equiv. of NCS dissolved in DMF for 3.5 min. Subsequently, the treatment with NCS was repeated for the same time interval. HPLC analysis of the crude obtained after cleavage of the NCS-treated peptidyl resin showed (Figure 1) the presence of cyclized oxytocin with ≥87% purity and no trace of the starting material, i.e., linear oxytocin [1,6-Cys(Acm)] (Figure 1 and Figures S3 and S4 in Supplementary Materials). Being an effective chlorinating agent, NCS was expected to chlorinate the Acm-protected Cys thiol, forming active species like halosulfonium cation and sulfenyl chloride, ultimately forming the disulfide bond with the release of Acm and HCl [27]. This result clearly demonstrated that the iterative treatments with a small amount of NCS were able to simultaneously remove the Acm groups from Cys thiols and form the desired disulfide bond on resin within a short timeframe.

**Figure 2:**
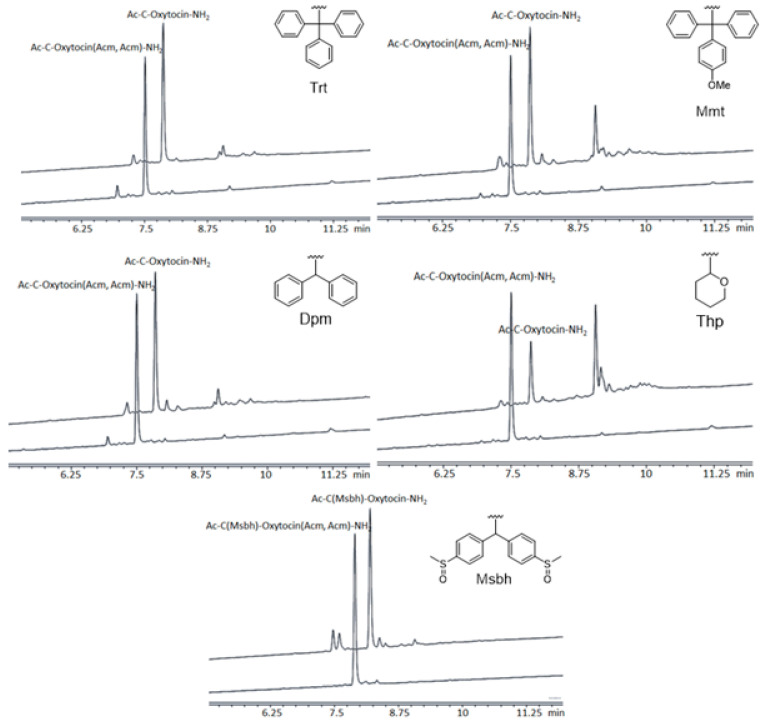
HPLC chromatograms of Ac-Cys(PG)-Oxytocin [1,6-Cys(Acm)]-NH_2_ and Ac-Cys(PG)-Oxytocin-NH_2_ obtained after NCS treatments (PG = Trt, Mmt, Dpm, Thp, and Msbh).

This success prompted us to investigate the NCS-mediated on-resin cyclization in the presence of other Cys thiol PGs. It is essential to validate the stability of other Cys PGs while using NCS so that those PGs can be used in combination with Acm during the synthesis of multi-disulfide peptides. We investigated five widely used Cys thiol PGs—Trt, Mmt, Dpm, Thp (tetrahydropyranyl), and Msbh (4,4′-dimethylsulfinylbenzhydryl). All of these PGs except Msbh were acid labile. Using those five Cys thiol PGs, we synthesized five batches of 10-mer Ac-Cys(PG)-Cys(Acm)-Tyr(*t*Bu)-Ile-Gln(Trt)-Asn(Trt)-Cys(Acm)-Pro-Leu-Gly-NH-Rink amide AM resin, where PG = Trt, Mmt, Dpm, Thp, and Msbh. To accommodate the Cys thiol PGs under experimentation, a Cys(PG) residue was included at the *N*-terminal of the linear oxytocin resin of each batch after peptide chain elongation following standard Fmoc/*t*Bu SPPS protocols. After the synthesis of the desired sequence, the *N*-terminal of each peptide chain was acetylated. The syntheses were confirmed by cleaving a small amount of peptidyl resin from each batch and then analyzing the crudes using HPLC and LCMS (Figure 2 and Figures S5–S9 in Supplementary Materials). Then, the peptidyl resin from each batch was treated with two consecutive treatments of NCS in DMF, with 3 equiv. of NCS used for 3.5 min in each treatment. Analysis of the peptidyl crudes after NCS treatments is presented in Figure 2.

From the results, it was evident that the use of NCS prompted the formation of cyclized Ac-Cys(PG)-oxytocin-NH-Rink amide resin in all cases (Figure 2 and Figures S10, S12, S14, S16 and S18 in Supplementary Materials). However, the extent of formation of the cyclized peptide varied widely across the Cys thiol PGs used. The HPLC purity of the cyclized peptide obtained from the Trt-, Mmt-, Dpm-, Thp-, and Msbh-protected linear peptides was 76.1%, 48.1%, 67.1%, 25.4%, and 68.4%, respectively. From this analysis, it was clear that the decreasing order of NCS treatment tolerance of Cys thiol PGs was Trt > Msbh > Dpm > Mmt > Thp. This was supported by the formation of disulfide-based side products at an HPLC retention time (RT) of 9.06 min (Figures S11, S13, S15 and S17 in Supplementary Materials). The percentage formation of these side products followed the opposite trend of the order observed in NCS treatment tolerance i.e., Thp (51.79%) > Mmt (26.36%) > Dpm (9.42%) > Trt (9.3%). Among acid-labile Cys PGs, the percentage formation of side products was at its maximum in the Thp-protected peptidyl resin and was at its minimum in the Trt-protected peptidyl resin. Overall, the Trt-protected resin was the best performing of all. The performance of the Dpm-protected peptidyl resin was moderately good—better than the Mmt-protected resin but inferior to the Trt-protected resin. In this sense, the Msbh-protected resin (Figure 2 and Figures S18 and S19 in Supplementary Materials) was similar to that of the Dpm-protected one.

**Figure 3:**
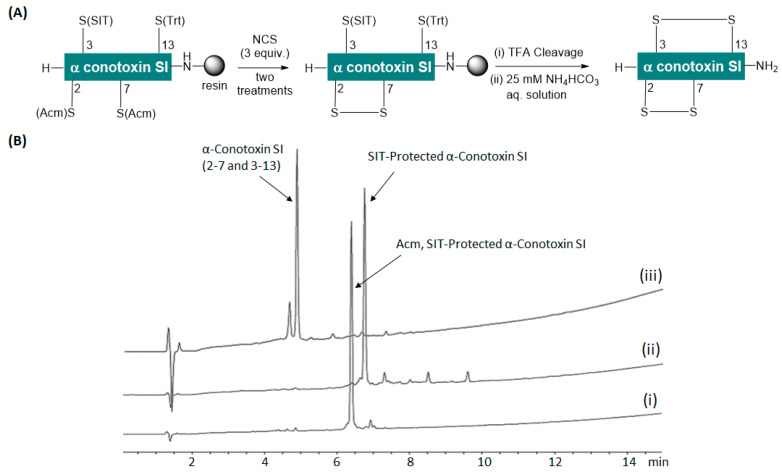
(**A**) Synthesis scheme and (**B**) HPLC chromatograms of (iii) cyclized α conotoxin SI from (i) Acm(Cys2 and Cys7) and SIT(Cys3)-protected α conotoxin SI and (ii) SIT(Cys3)-protected α conotoxin SI.

Finally, we attempted the synthesis of 13-mer α-conotoxin SI (H-ICCNPACGPKYSC-NH_2_). The peptide contains two disulfide bonds between Cys2-Cys7 and Cys3-Cys13. The conotoxin was synthesized on Fmoc-Rink amide AM resin following Fmoc-SPPS protocols. During the synthesis, Fmoc-Cys(Acm)-OH was used to incorporate the Cys residues at positions 2 and 7, while Fmoc-Cys(SIT)-OH and Fmoc-Cys(Trt)-OH were used to incorporate Cys residues at positions 3 and 13, respectively (Figure 3B(i) and Figure S22 in Supplementary Materials). To cyclize α-conotoxin SI, the disulfide bond between Cys2 and Cys7 was first intended to form on resin, followed by the formation of the second disulfide bond (Cys3-Cys13) in solution using the SIT-directed method. It is worth mentioning that the stability of the Cys(SIT) residue against the NCS-mediated on-resin removal of Acm was validated in a similar fashion as that of Trt mentioned earlier (Figures S1, S20 and S21 in Supplementary Materials). After synthesis, the protected linear α-conotoxin SI resin was treated with two successive treatments of NCS (3 equiv.), with each treatment lasting for 3.5 min. Following the NCS treatment, the peptidyl resin was cleaved to yield the SIT-protected conotoxin, and a small portion of the crude product was analyzed to confirm the formation of the first disulfide bond (Cys2-Cys7) on resin. Figure 3B(ii) and Figure S23 in Supplementary Materials show the formation of the first disulfide, with an HPLC purity of 81.7%. Therefore, the use of NCS enabled efficient on-resin removal of Acm and simultaneous disulfide formation, contrary to the conventional practice of removing Acm in the final step of multi-disulfide peptide synthesis. These results confirmed the compatibility of the Acm removal with NCS with Trt and SIT protection of other Cys present in the sequence. Subsequently, we attempted the formation of the second disulfide bond (Cys3-Cys13) by dissolving the SIT-protected crude peptide in a 25 mM aqueous solution of NH_4_HCO_3_ (pH ≤ 8), stirring vigorously and monitoring the reaction progress using HPLC and LCMS. Figure 3B(iii) and Figure S24 in Supplementary Materials demonstrate the successful formation of the second disulfide bond, confirming the synthesis of fully cyclized α-conotoxin SI. The SIT-directed disulfide formation was rapid and achieved within 30 min. The cyclized conotoxin with native disulfide linkages (Cys2-Cys7 and Cys3-Cys13) was the major product (83.1% HPLC purity), along with a misfolded isomer (14.3% HPLC purity). This result was in accordance with our previous attempts to cyclize linear α-conotoxin SI using different methods. The formation of the misfolded isomer in the final stage of conotoxin cyclization was expected and previously reported by several groups, including ours [32,33,34].

## 3. Materials and Methods

### 3.1. General

All Fmoc-L-amino acids, including Fmoc-Cys(SIT)-OH, Fmoc-Cys(Acm)-OH, and Fmoc-Rink amide AM resin (loading 0.64 mmol/g), were purchased from Iris Biotech GmbH, Marktredwitz, Germany. *N,N′*-Diisopropylcarbodiimide (DIC) was purchased from Sisco Research Laboratories Pvt. Ltd., Mumbai, India. OxymaPure were obtained as gifts from Luxembourg Industries Ltd., Tel Aviv, Israel. All other common solvents and reagents used in this study were purchased from a well-known commercial supplier like Merck, Rahway, NJ, USA. An Agilent 1100 system fitted with a Phenomenex Aeris^TM^C18 column (3 μm, 4.6 × 150 mm), a flow rate of 1.0 mL/min, and a UV detection of 220 nm were used for analytical HPLC. All samples in HPLC were analyzed over a gradient of 5–95% CH_3_CN (0.1% TFA)/H_2_O (0.1% TFA) for 15 min unless otherwise mentioned. Chemstation software (version B.02.01 SR1) was used to analyze the data obtained from HPLC. Mass analysis was performed using a Thermo Fischer Scientific Inc. (Waltham, MA, USA) manufactured UltiMate 3000 UHPLC-ISQ EC single quadrupole mass spectrometer in positive-ion mode over a 5–95% gradient of CH_3_CN (0.1% HCOOH)/H_2_O (0.1% HCOOH) for 15 min unless otherwise specified. Data from the mass spectrometer were analyzed using Chromeleon^TM^ (CDS) Software (version 7.3).

### 3.2. Solid-Phase Peptide Synthesis

All peptides were synthesized following standard Fmoc/*t*Bu-based SPPS protocols with a 0.1–0.2 mmol scale. The synthesis was performed on Fmoc-Rink amide AM resin (0.64 mmol/g) manually in polypropylene syringes fitted with a polyethylene porous disc. At first, the resin was washed using dimethylformamide (DMF, 1 mL, 3 × 1 min), dichloromethane (DCM, 1 mL, 3 × 1 min), and DMF (1 mL, 3 × 1 min). After that, Fmoc deprotection was achieved using 20% piperidine in DMF (1 × 1 min and 1 × 7 min), followed by washing (DMF, 1 mL, 3 × 1 min). In general, Fmoc-amino acids (3 equiv.) were incorporated using DIC (3 equiv) and OxymaPure (3 equiv.) in DMF for 40–45 min at rt. Afterwards, the resin was washed (DMF, 1 mL, 3 × 1 min). This was repeated until the final sequence was achieved. Fmoc from the last coupled amino acid was removed following the procedure mentioned above. After removing Fmoc, peptidyl resins were washed (DMF, 1 mL, 3 × 1 min and DCM, 1 mL, 3 × 1 min) and dried.

Cleavage and global deprotection of the peptidyl resin were performed with TFA/TIS/H_2_O (95:2.5:2.5) for 45 min–1 h at rt. After cleavage, the mixture was filtered and the peptide was precipitated with chilled Et_2_O. Next, it was centrifuged and decanted. This was repeated three times. Finally, the peptide obtained was dissolved in H_2_O, lyophilized, and analyzed by HPLC and LCMS.

### 3.3. Cyclization of Acm-Protected Peptides on Resin

A total of 50 mg of Acm-protected peptidyl resin were treated with 3 equiv. of NCS dissolved in DMF for 3.5 min. The treatment was carried out twice. After NCS treatment, the peptidyl resin was washed, dried, and cleaved, and the crude obtained was analyzed using HPLC and LCMS.

### 3.4. Cyclization of SIT-Protected Peptides in Solution

The SIT-protected crude peptide was dissolved in H_2_O (0.5–1 mg/mL) with a few drops of CH_3_CN and stirred vigorously at rt. The pH was maintained around 8 using 25 mM of aqueous solution of NH_4_HCO_3_. The cyclization was monitored timewise using HPLC and LCMS.

## 4. Conclusions

Apart from bioconjugation, the ability of Cys residues to form a disulfide bond has been central to its utility in most peptidyl drug candidates. Cys(Acm) is a ready-to-use Cys residue that enables efficient disulfide synthesis with multimodal removal conditions and compatibility with most other Cys derivatives. However, the harsh removal conditions of Acm from Cys thiol always poses a challenge. In the present work, we demonstrated a fast alternative method using NCS for Acm removal on resin. The method is straightforward in the sense that it removes reaction by-products using simple filtration. This is advantageous compared to conventional methods of oxidizing Cys(Acm) residues, such as the use of iodine. However, like iodine, Met- and Trp-containing peptides show sensitivity towards NCS treatments [35]. Additionally, the NCS-mediated on-resin oxidation of Cys(Acm) residues was compatible with the use of other Cys thiol PGs such as Trt and SIT. Interestingly, NCS does not remove the Trt group, which is removed with iodine. Ultimately, all of these advantages culminated in the successful synthesis of a multi-disulfide peptide like α-conotoxin SI. This strategy (two Cys protected with Acm and one with Trt) could be also used for the synthesis of peptides containing a disulfide bridge and a Cys, which later can be used for bioconjugation through the free thiol liberated after TFA treatment of the Trt-containing peptide.

## Data Availability

Data are contained within the article and Supplementary Materials.

## Supplementary Materials

The following supporting information can be downloaded at: https://www.mdpi.com/article/10.3390/ijms26062523/s1.
