# Supplementary material for: On-Resin Acetamidomethyl (Acm) Removal and Disulfide Formation in Cysteinyl Peptides Using N-Chlorosuccinimide (NCS) in the Presence of Other Cys-Protecting Groups
Source: Int J Mol Sci. 2025 Mar 11;26(6):2523. doi: 10.3390/ijms26062523 (PMC11941928; doi:10.3390/ijms26062523)
Supplement: Supplementary file 1 [file ijms-26-02523-s001.zip › ijms-3502186-supplementary.pdf]

Supporting Information

for

On-Resin Acetamidomethyl (Acm) Removal and Disulfide Formation in Cysteinyl Peptides Using *N*-Chlorosuccinimide (NCS) in the Presence of Other Cys-Protecting Groups

Amit Chakraborty [1], Fernando Albericio [1,2,*] and Beatriz G. de la Torre [1,3,*]

[1] Peptide Science Laboratory, School of Chemistry and Physics, University of KwaZulu-Natal, Westville, Durban 4000, South Africa; amit24816@gmail.com

[2] Department of Organic Chemistry, University of Barcelona, 08028 Barcelona, Spain

[3] School of Laboratory Medicine and Medical Sciences, College of Health Sciences, University of KwaZulu-Natal, Durban 4041, South Africa

* Correspondence: albericio@ukzn.ac.za (F.A.); garciadelatorreb@ukzn.ac.za (B.G.d.l.T.)

HPLC Data:

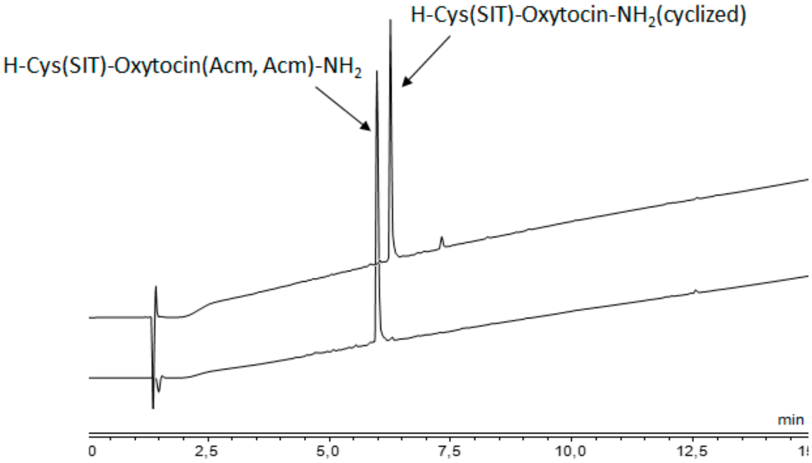

Figure S1. HPLC chromatograms of H-Cys(SIT)-oxytocin[1,6-Cys(Acm)]-NH_2 and H-Cys(SIT)-oxytocin-NH_2 obtained after NCS treatments.

LCMS Data:

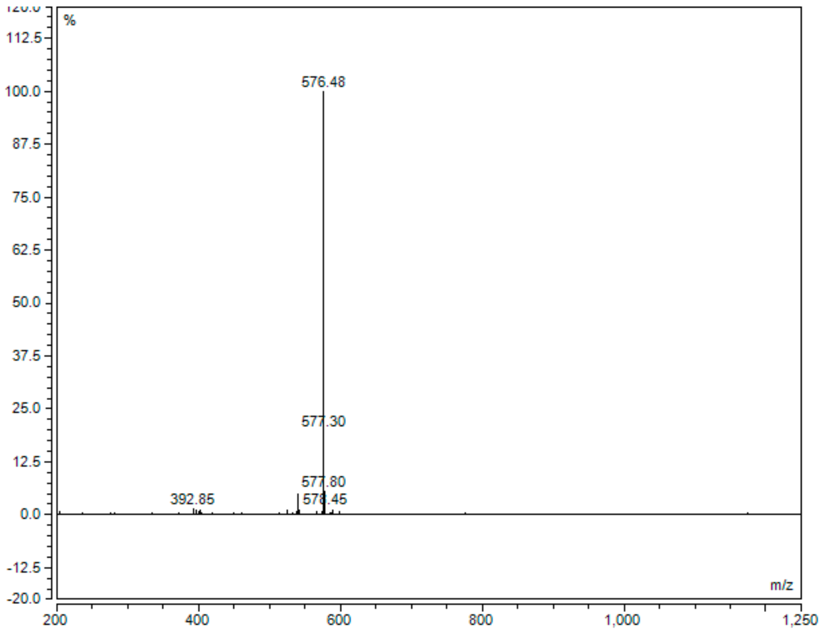

Figure S2. LCMS of H-oxytocin(Acm, Acm)-NH_2; m/z calculated as 576.27 ($M+2H^+/2$).

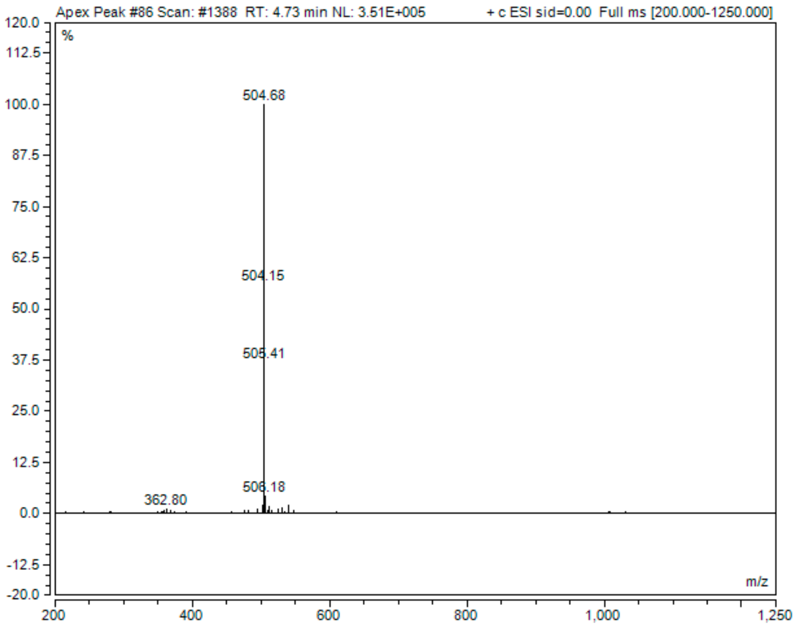

Figure S3. LCMS of cyclized H-oxytocin-NH2; m/z calculated as 504.23 (M+2H$^+$/2).

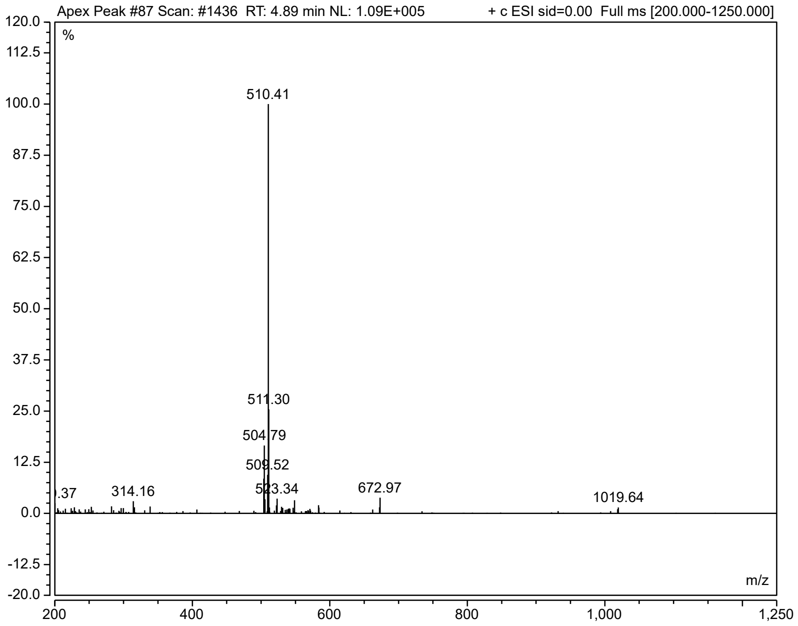

Figure S4. LCMS of the major side product during cyclization of the protected H-oxytocin-NH resin.

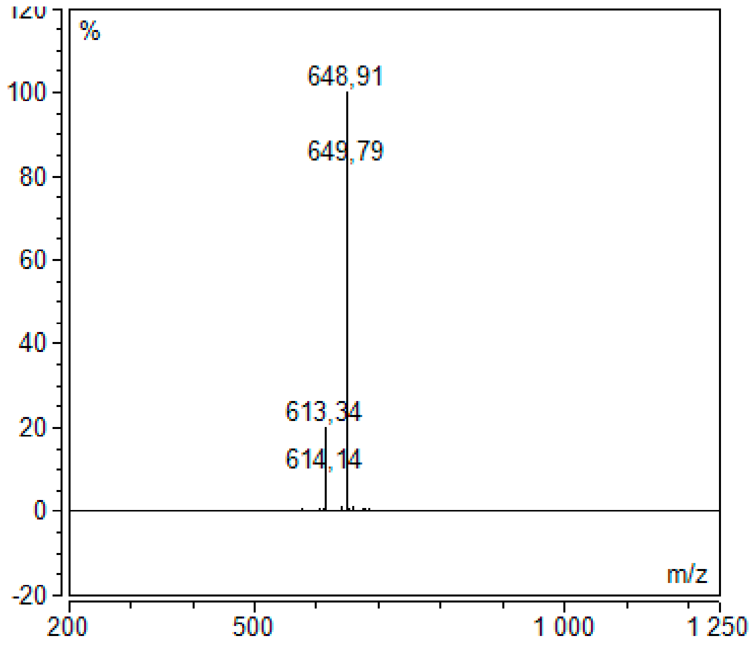

Figure S5. LCMS of Ac-Cys-oxytocin(Acm, Acm)-NH_2 obtained after cleaving Ac-Cys(Dpm)-Cys(Acm)-Tyr(tBu)-Ile-Gln(Trt)-Asn(Trt)-Cys(Acm)-Pro-Leu-Gly-NH-Rink amide AM resin; m/z calculated as 648.78 ($M+2H^+/2$).

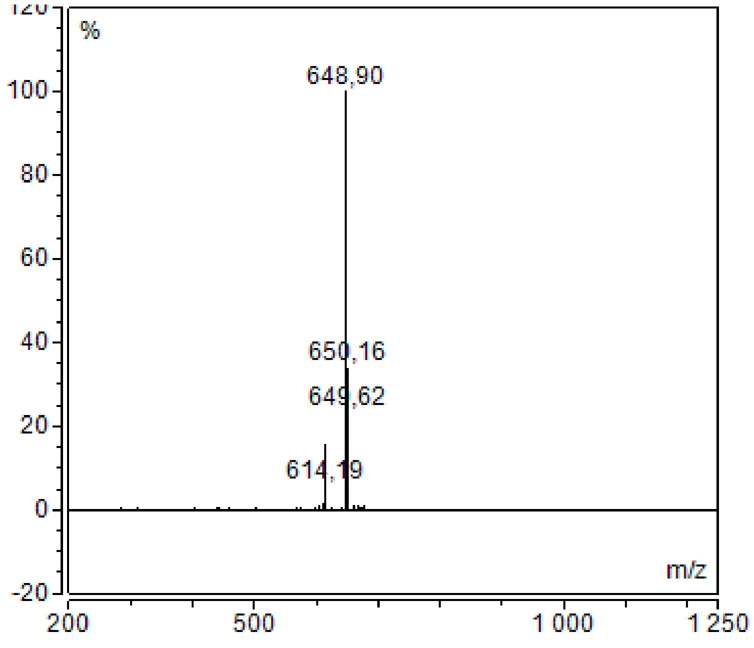

Figure S6. LCMS of Ac-Cys-oxytocin(Acm, Acm)-NH_2 obtained after cleaving Ac-Cys(Mmt)-Cys(Acm)-Tyr(tBu)-Ile-Gln(Trt)-Asn(Trt)-Cys(Acm)-Pro-Leu-Gly-NH-Rink amide AM resin; m/z calculated as 648.78 ($M+2H^+/2$).

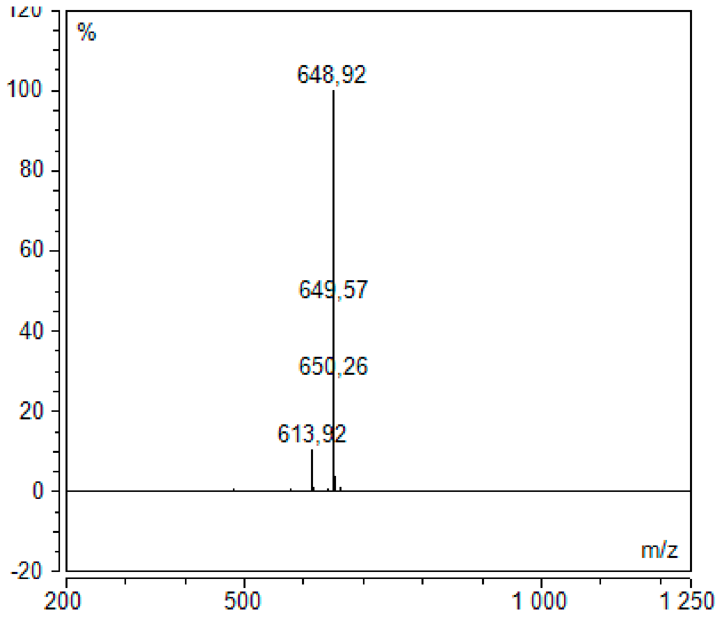

Figure S7. LCMS of Ac-Cys-oxytocin(Acm, Acm)-NH_2 obtained after cleaving Ac-Cys(Thp)-Cys(Acm)-Tyr(tBu)-Ile-Gln(Trt)-Asn(Trt)-Cys(Acm)-Pro-Leu-Gly-NH-Rink amide AM resin; m/z calculated as 648.78 (M+2H^+/2).

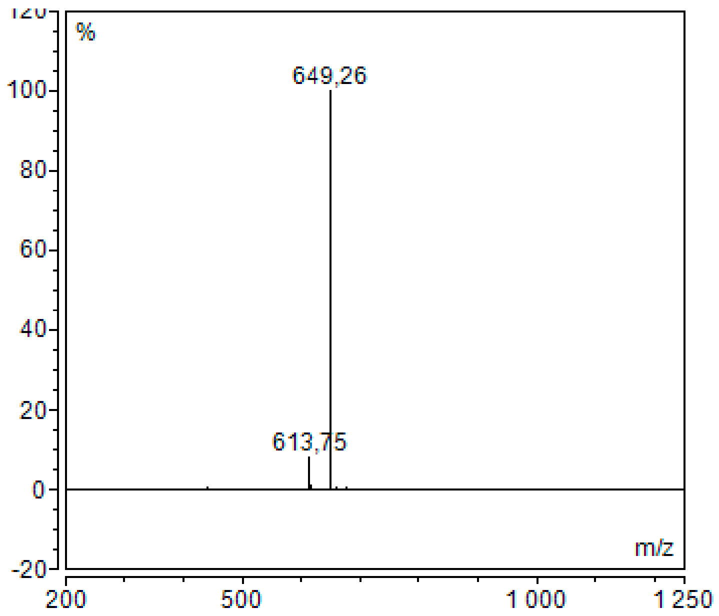

Figure S8. LCMS of Ac-Cys-oxytocin(Acm, Acm)-NH_2 obtained after cleaving Ac-Cys(Trt)-Cys(Acm)-Tyr(tBu)-Ile-Gln(Trt)-Asn(Trt)-Cys(Acm)-Pro-Leu-Gly-NH-Rink amide AM resin; m/z calculated as 648.78 (M+2H^+/2).

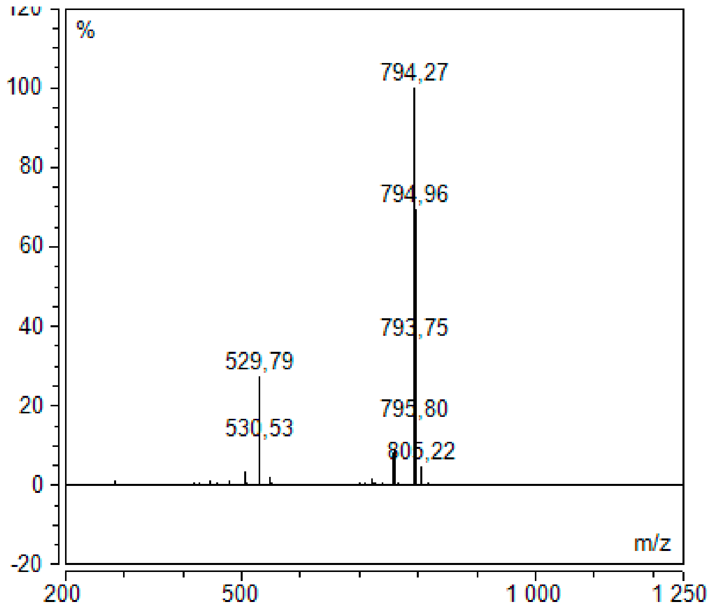

Figure S9. LCMS of Ac-Cys(Msbh)-oxytocin(Acm, Acm)-NH_2 obtained after cleaving Ac-Cys(Msbh)-Cys(Acm)-Tyr(tBu)-Ile-Gln(Trt)-Asn(Trt)-Cys(Acm)-Pro-Leu-Gly-NH-Rink amide AM resin; m/z calculated as 793.8 ($M+2H^+/2$), 529.54 ($M+3H^+/3$).

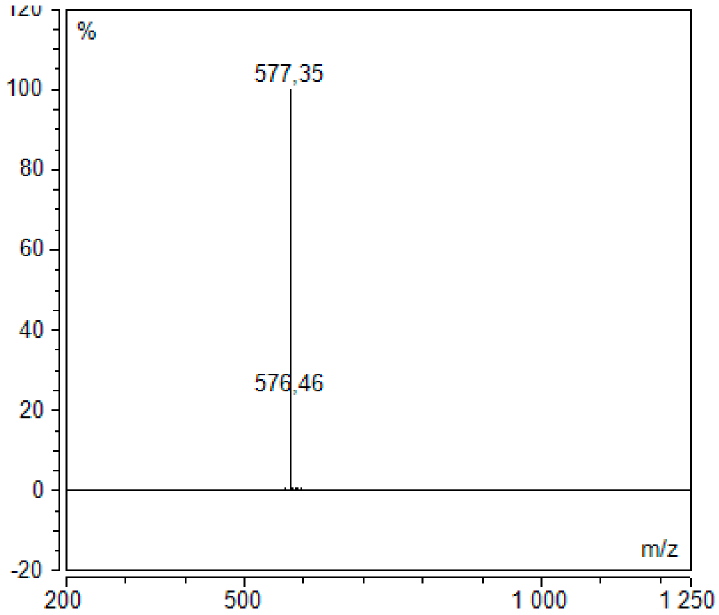

Figure S10. LCMS of cyclized Ac-Cys-oxytocin-NH_2 obtained after on-resin cyclization of Ac-Cys(Dpm)-Cys(Acm)-Tyr(tBu)-Ile-Gln(Trt)-Asn(Trt)-Cys(Acm)-Pro-Leu-Gly-NH-Rink amide AM resin using NCS; m/z as calculated 576.74 ($M+2H^+/2$).

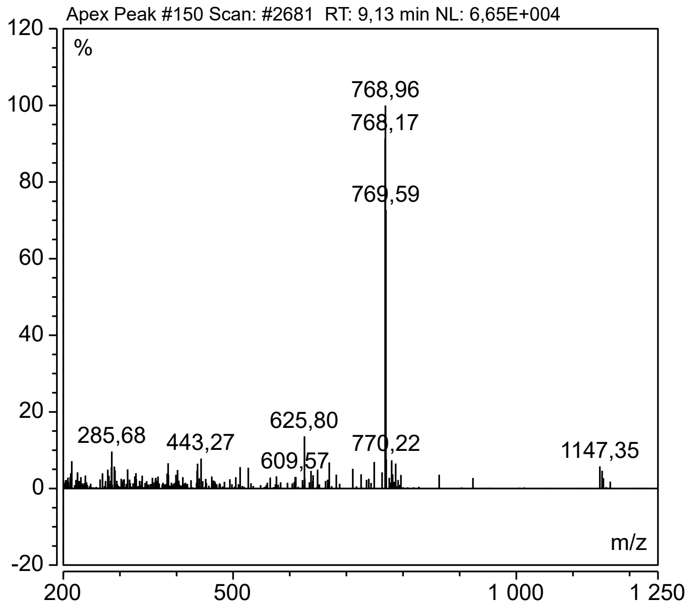

Figure S11. LCMS of major side product obtained after on-resin cyclization of Ac-Cys(Dpm)-Cys(Acm)-Tyr(tBu)-Ile-Gln(Trt)-Asn(Trt)-Cys(Acm)-Pro-Leu-Gly-NH-Rink amide AM resin using NCS.

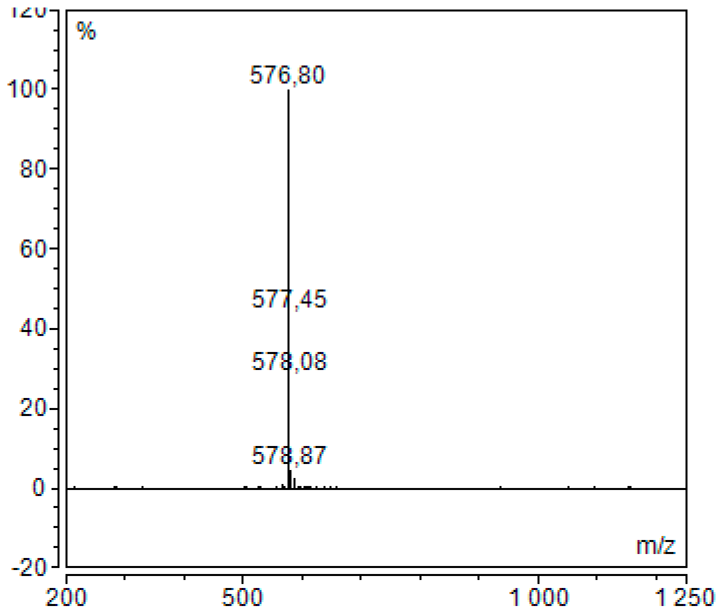

Figure S12. LCMS of cyclized Ac-Cys-oxytocin-NH_2 obtained after on-resin cyclization of Ac-Cys(Mmt)-Cys(Acm)-Tyr(tBu)-Ile-Gln(Trt)-Asn(Trt)-Cys(Acm)-Pro-Leu-Gly-NH-Rink amide AM resin using NCS; m/z calculated as 576.74 ($M+2H^+/2$).

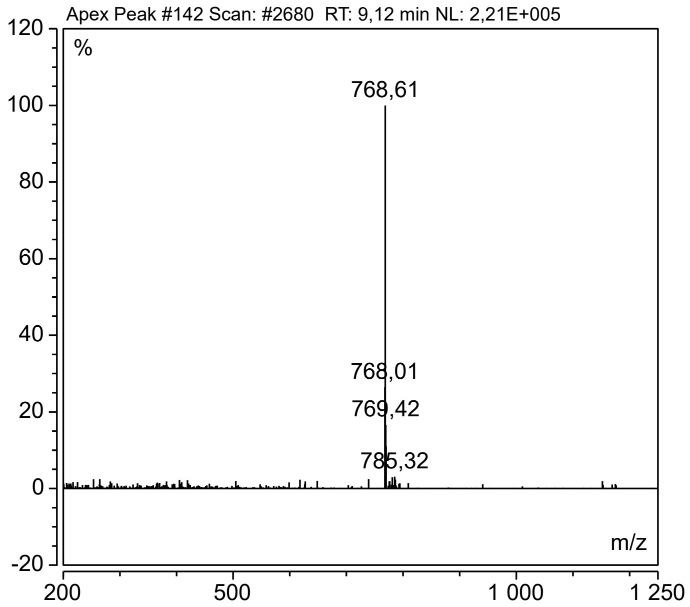

Figure S13. LCMS of major side product obtained after on-resin cyclization of Ac-Cys(Mmt)-Cys(Acm)-Tyr(tBu)-Ile-Gln(Trt)-Asn(Trt)-Cys(Acm)-Pro-Leu-Gly-NH-Rink amide AM resin using NCS.

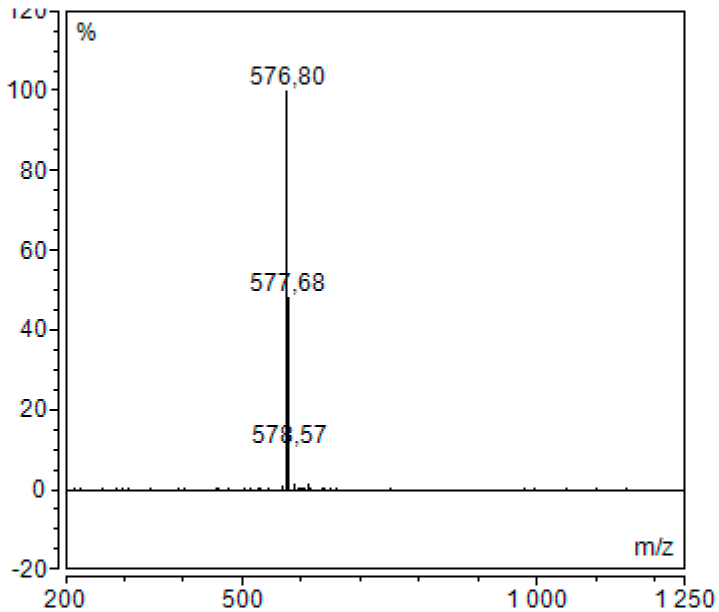

Figure S14. LCMS of cyclized Ac-Cys-oxytocin-NH_2 obtained after on-resin cyclization of Ac-Cys(Thp)-Cys(Acm)-Tyr(tBu)-Ile-Gln(Trt)-Asn(Trt)-Cys(Acm)-Pro-Leu-Gly-NH-Rink amide AM resin using NCS; m/z calculated as 576.74 ($M+2H^+/2$).

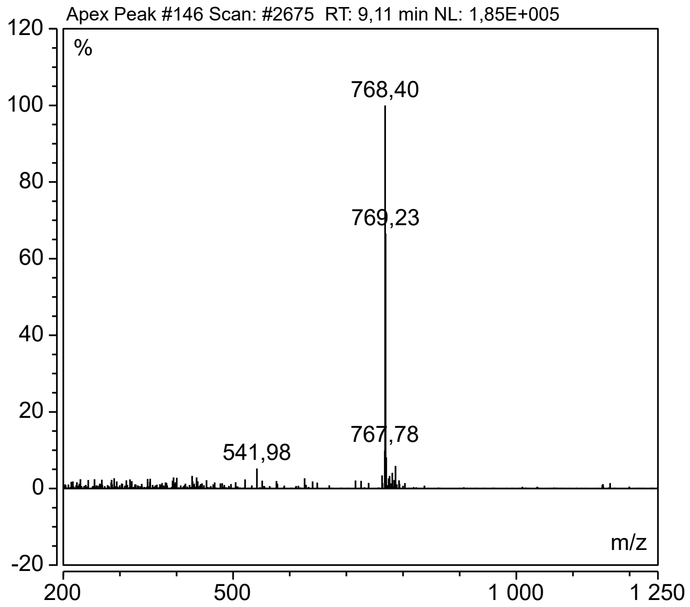

Figure S15. LCMS of major side product obtained after on-resin cyclization of Ac-Cys(Thp)-Cys(Acm)-Tyr(tBu)-Ile-Gln(Trt)-Asn(Trt)-Cys(Acm)-Pro-Leu-Gly-NH-Rink amide AM resin using NCS.

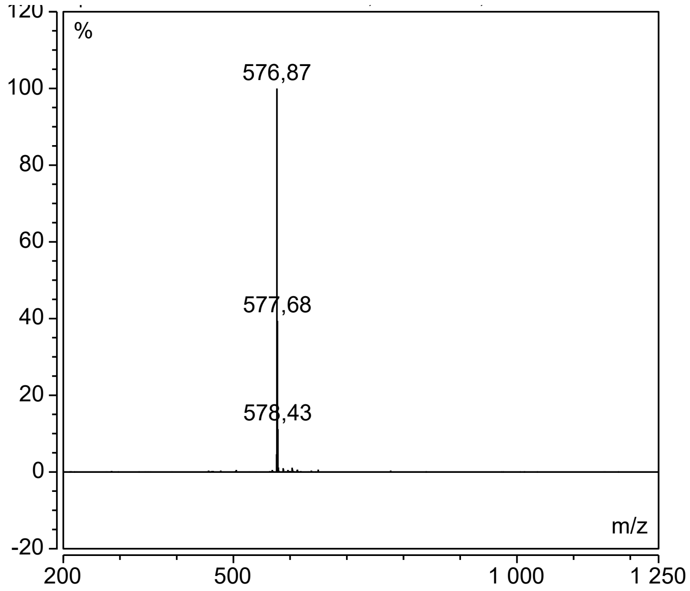

Figure S16. LCMS of cyclized Ac-Cys-oxytocin-NH_2 obtained after on-resin cyclization of Ac-Cys(Trt)-Cys(Acm)-Tyr(tBu)-Ile-Gln(Trt)-Asn(Trt)-Cys(Acm)-Pro-Leu-Gly-NH-Rink amide AM resin using NCS; m/z calculated as 576.74 ($M+2H^+/2$).

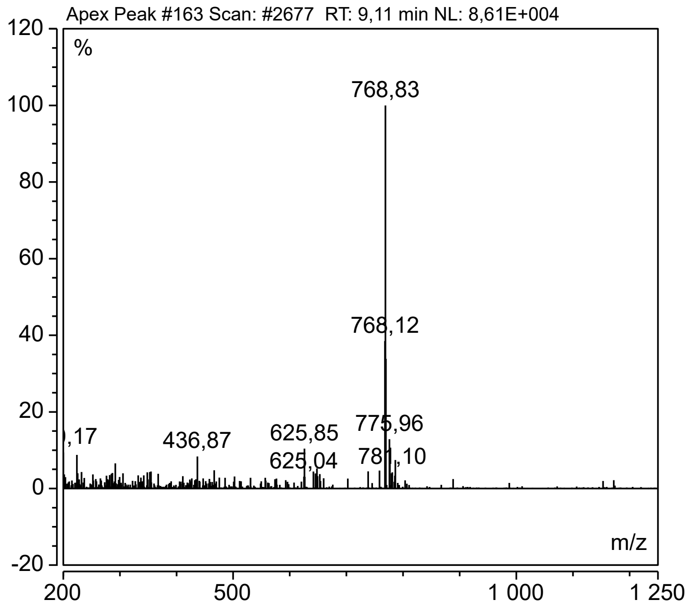

Figure S17. LCMS of major side product obtained after on-resin cyclization of Ac-Cys(Trt)-Cys(Acm)-Tyr(tBu)-Ile-Gln(Trt)-Asn(Trt)-Cys(Acm)-Pro-Leu-Gly-NH-Rink amide AM resin using NCS.

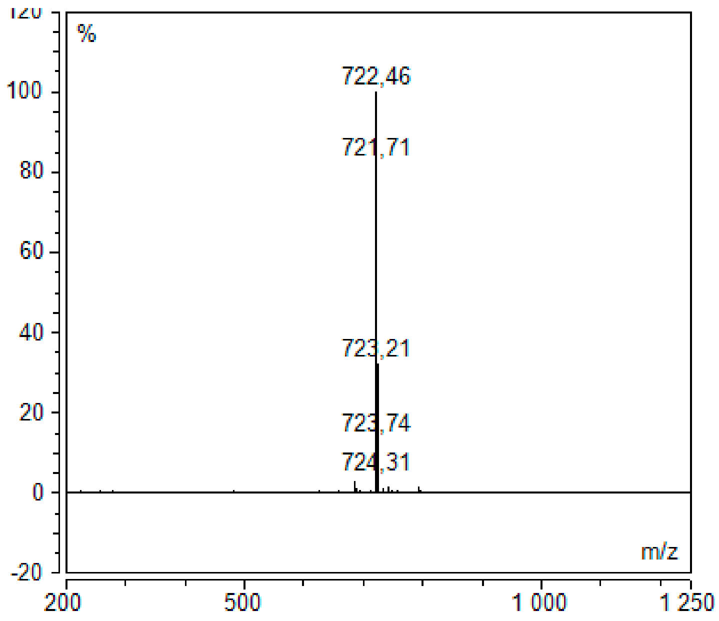

Figure S18. LCMS of cyclized Ac-Cys(Msbh)-oxytocin-NH_2 obtained after on-resin cyclization of Ac-Cys(Msbh)-Cys(Acm)-Tyr(tBu)-Ile-Gln(Trt)-Asn(Trt)-Cys(Acm)-Pro-Leu-Gly-NH-Rink amide AM resin using NCS; m/z calculated as 721.76 (M+2H^+/2).

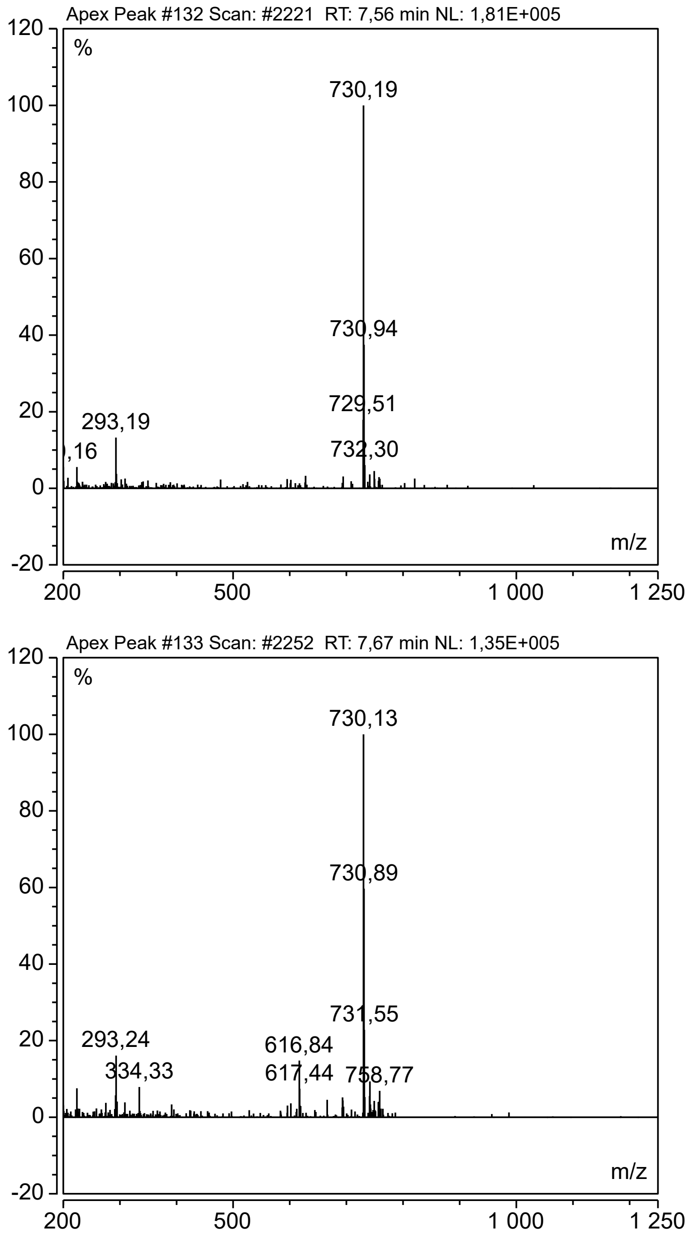

Figure S19. LCMS of major side products obtained after on-resin cyclization of Ac-Cys(Msbh)-Cys(Acm)-Tyr(tBu)-Ile-Gln(Trt)-Asn(Trt)-Cys(Acm)-Pro-Leu-Gly-NH-Rink amide AM resin using NCS.

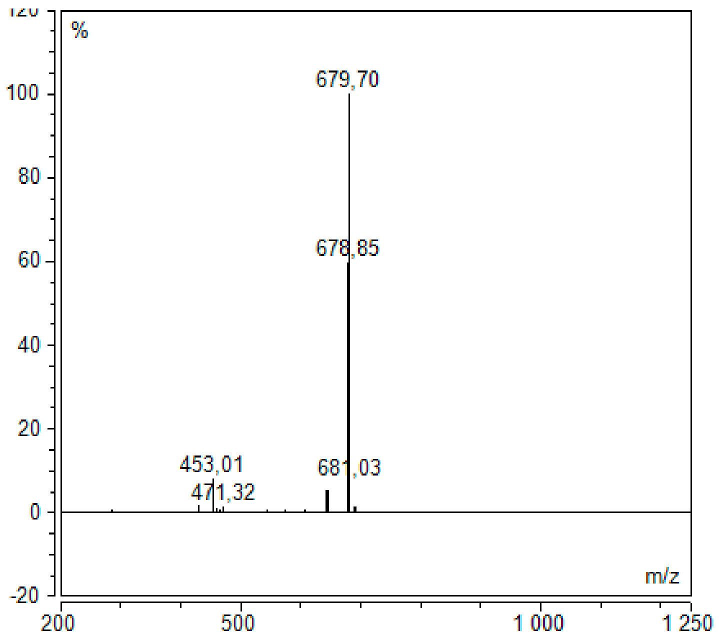

Figure S20. LCMS of H-Cys(SIT)-oxytocin[1,6 Cys(Acm)]-NH_2; m/z calculated as 678.80 ($M+2H^+/2$), 452.87 ($M+3H^+/3$).

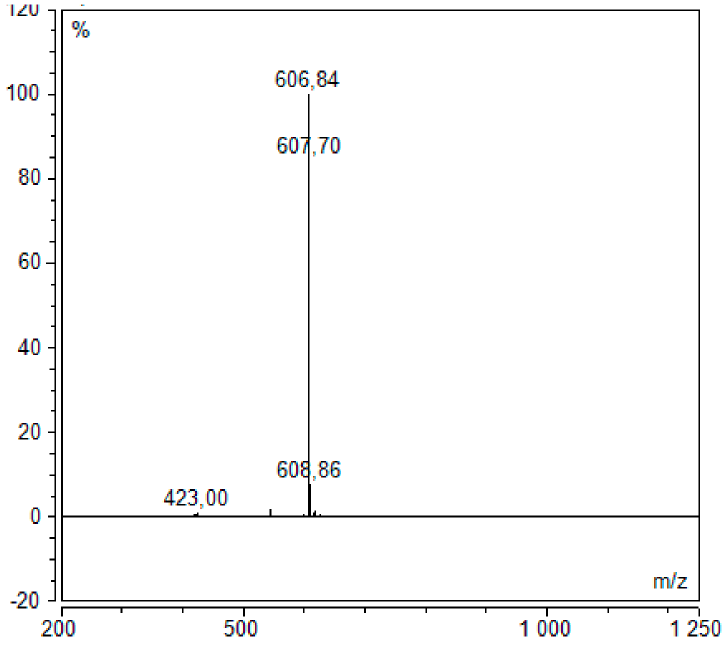

Figure S21. LCMS of cyclized H-Cys(SIT)-oxytocin-NH_2; m/z calculated as 606.76 ($M+2H^+/2$).

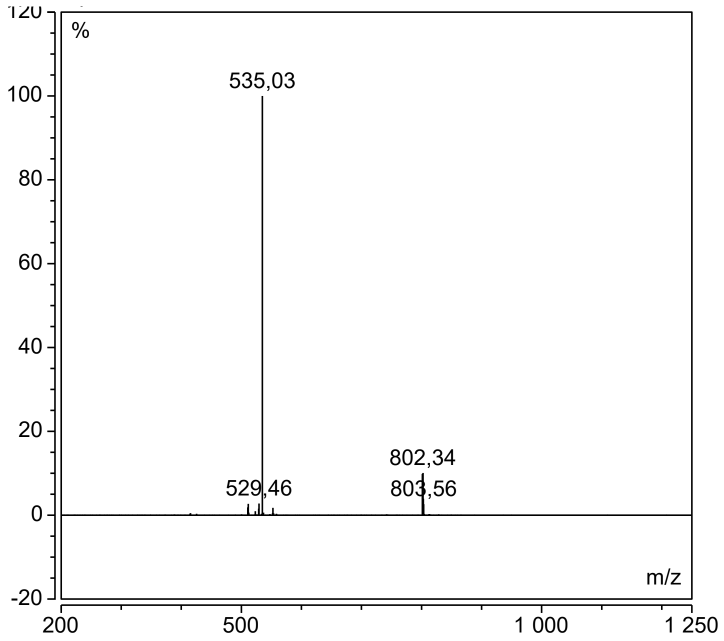

Figure S22. LCMS of H-IC(Acm)C(SIT)NPAC(Acm)GPKYSC-NH_2; m/z calculated as 802.01 ($M+2H^+/2$), 535.01 ($M+3H^+/3$).

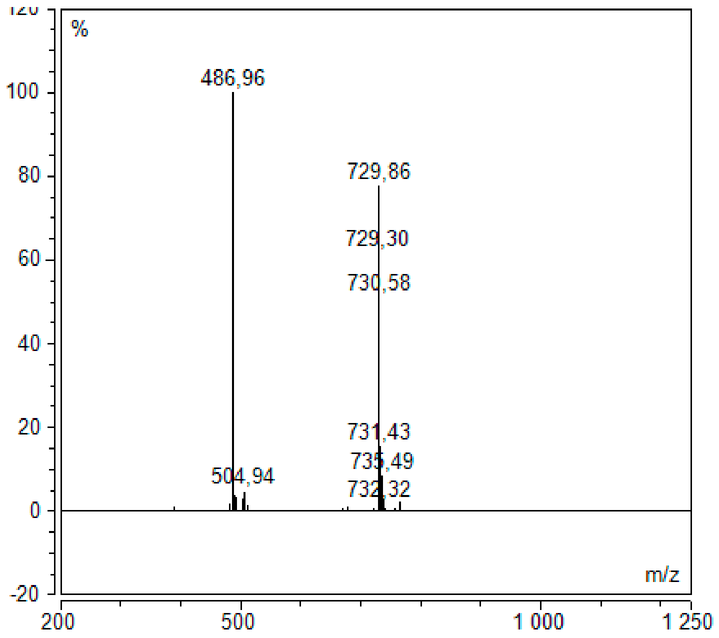

Figure S23. LCMS of cyclized H-ICC(SIT)NPACGPKYSC-NH_2 (SS, Cys2-Cys7) with one disulfide bond; m/z calculated as 729.30 ($M+2H^+/2$), 486.20 ($M+3H^+/3$).

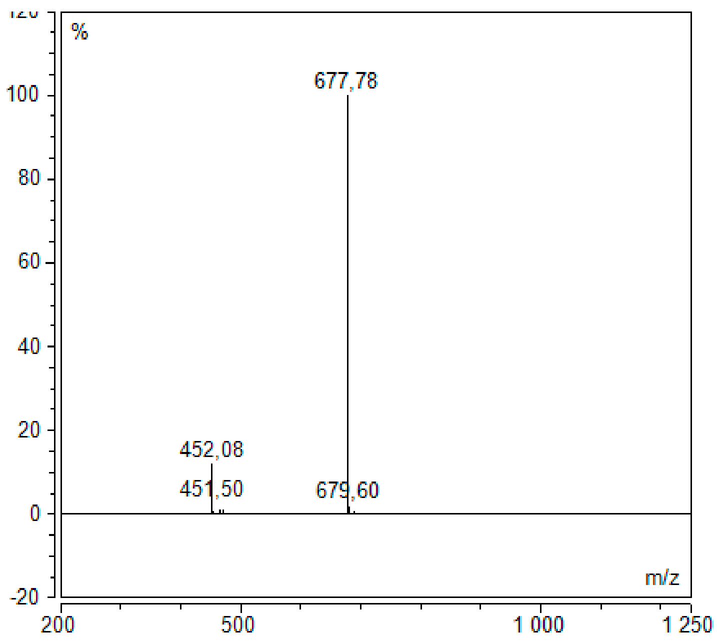

Figure S24. LCMS of fully cyclized H-ICCNPACGPKYSC-NH_2 (2SS, Cys2-Cys7 and Cys3-Cys13) with one disulfide bond; m/z calculated as 677.27 (M+2H^+/2), 451.85 (M+3H^+/3).
